# A new trauma severity scoring system adapted to wearable monitoring: A pilot study

**DOI:** 10.1371/journal.pone.0318290

**Published:** 2025-03-04

**Authors:** Alice Lemarquand, Pierre Jannot, Léo Kammerlocher, Gaëlle Lissorgues, Michel Behr, Pierre-Jean Arnoux, Salah Boussen

**Affiliations:** 1 Laboratoire de Biomécanique Appliquée, Université Gustave Eiffel, Aix-Marseille Université, Marseille, France; 2 Laboratoire ESYCOM, Université Gustave Eiffel, Noisy-le-Grand, France; 3 Ecole National Supérieure des Officiers Sapeurs-Pompiers, Aix-en-Provence, France; 4 Intensive Care and Anesthesiology Department, La Timone Teaching Hospital, Aix-Marseille Université, Assistance Publique Hôpitaux de Marseille, Marseille, France; 5 Anesthesiology and Intensive Care Unit, Sainte Anne National Military Teaching Hospital, Toulon, France; Kaohsuing Medical University Hospital, TAIWAN

## Abstract

Wearable technologies represent a strong development axis for various medical applications and these devices are increasingly used in daily life as illustrated by smart watches’ popularisation. Combined with new data processing methods, it constitutes a promising opportunity for telemonitoring, triage in mass casualty situations, or early diagnosis after a traffic or sport accident. An approach to processing the physiological data is to develop severity scoring systems to quantify the critical level of an individual’s health status. However, the existing severity scores require a human evaluation. A first version of a severity scoring system adapted to continuous and real-time wearable monitoring is proposed in this article. The focus is made on three physiological parameters straightforwardly measurable with wrist-wearables: heart rate, respiratory rate, and SpO_2_, which may be enough to characterise continuously hemodynamic and respiratory status. Intermediate score functions corresponding to each physiological parameter have been established using a sigmoid model. The boundary conditions have been defined based on a survey conducted among 54 health professionals. An adapted function has also been developed to merge the three intermediate scores into a global score. The scores are associated with a triage tricolour code: green for a low-priority casualty, orange for a delayable one, red for an urgent one. Preliminary confrontation of the new severity scoring system with real data has been carried out using a database of 84 subjects admitted to the intensive care unit. Colour classification by the new scoring system was compared with independent physicians’ direct evaluation as a reference. The prediction success rate values 74% over the entire database. Two examples of continuous monitoring over time are also given. The new score has turned out to be consistent, and may be easily upgraded with the integration of additional vital signs monitoring or medical information.

## Introduction

The assessment of severity levels in illnesses or injuries is crucial for determining appropriate pre-hospital and in-hospital care. Numerous scoring systems have been developed to aid health professionals in this evaluation [1–11]. These severity scores are also instrumental in mortality prediction, serving as valuable epidemiological tools. For example, they are relevant in comparing care structures or systems. Moreover, in mass casualty incident (MCI) situations such as pandemics, large-scale accidents, natural disasters, and terrorist or military crises, scoring systems are vital. In such scenarios, where medical resources are stretched thin, efficient triage becomes imperative to optimise care management by prioritizing casualties. Recent years have witnessed a paradigm shift in emergency medicine, particularly in the context of MCIs. Traditional severity scoring systems, while effective, often fall short in dynamic and rapidly evolving mass casualty scenarios for which the ability to continuously monitor and assess the severity of injuries in real-time becomes crucial. This is where wearable technology steps in, bridging the gap with its capability for real-time, continuous data collection and transmission. Wearable devices, equipped with advanced sensors, offer a promising solution to the limitations of static severity scoring methods, enabling a more responsive and adaptive approach for emergency care. Indeed, the deployment of wearable technology can be transformative in MCI situations. Imagine a scenario where each injured individual is equipped with a wearable device that not only assesses its medical condition in real-time, but also provides its exact location. This would not only streamline the triage process but also enhance the coordination of pre-hospital and in-hospital care. Such technology could be instrumental in prioritizing care for the most severely injured, managing resources effectively, and ultimately saving lives. The real-time data provided by these wearables could also assist in creating a dynamic map of the incident, aiding in efficient resource allocation and response planning.

Among already-existing physiological severity scores, the Glasgow Coma Scale (GCS) measures a person’s consciousness level and is particularly useful in head injury cases [4,12,13]. It is computed by summing scores across three criteria: eye opening, motor response, and verbal response. The Early Warning Score (EWS) and its more recent variant, the Modified Early Warning Score (MEWS), have been developed to identify patients needing more intensive care. Similar to the GCS, they are calculated by aggregating scores from five criteria: systolic blood pressure (SPS), heart rate, respiratory rate, temperature, and the “Alert, Verbal, Pain, Unresponsive” (AVPU) score, akin to the GCS [14–17]. The Revised Trauma Score (RTS) is designed for prehospital evaluation and is a continuous function of GCS, SPS, and respiration rate [3]. In addition to physiological scores, anatomical score have also been developed, mainly based on the Abbreviated Injury Score (AIS) [18]. Combined systems like the Trauma and Injury Severity Score (TRISS) [19] and A Severity Characterization of Trauma (ASCOT) [11,20] integrate both anatomical and physiological aspects. However, these scoring systems require at least one step of human evaluation, preventing automatic calculation. This can be time-consuming, especially when assessing numerous individuals. Additionally, these scores are typically calculated at a single time point, which is not suitable for monitoring health status deterioration.

In the era of the Internet of Things, wearables present an opportunity for easy, fast, non-invasive, and continuous monitoring of vital signs. Utilizing electronic, optical, mechanical, or biochemical technologies, these sensors can measure environmental, motion, position, or physiological parameters [21–24]. They may include miniaturized sensors embedded in implants, accessories, garments, or skin-adherent systems like patches or tattoos. Wristbands and smartwatches, in particular, are becoming more widespread [25,26]. Wearable devices often employ wireless communication protocols to transmit data to smartphones or remote servers for online or offline processing [27–29]. The applications of these devices range from entertainment to well-being (gaming, sport tracking, activity or emotion recognition…), as well as in health such as biomedical research, remote telemonitoring of vulnerable individuals, mass casualty triage, or early diagnosis in traffic or sports accidents if the device is worn in advance. Indeed, wearable technologies offer a promising tool for health professionals to monitor an individual’s medical condition straightforwardly and continuously, for instance based on scoring methods or track and trigger systems [30]. They are valuable in care management optimisation, which can help shorten medical response times, reduce the severity of sequelae, and decrease mortality. Wearables represent a fast-growing sector, thanks to breakthroughs in miniaturised sensors, communication protocols, data processing and storing, power supplies, ergonomics, and integration. Taken together, these advances should enhance devices’ reliability and data quality, and lead to the emergence of more and more medical-grade devices [31,32].

Heart rate (HR), respiratory rate (RR), and peripheral oxygen saturation (SpO_2_) are often sufficient to reflect an individual’s hemodynamic and respiratory status. HR can be measured through electrocardiography (cardiac electrical activity measurement) [33,34], or photoplethysmography (PPG) (pulse wave measurement) [35,36]. PPG is an optical technique that detects blood circulation’s volumetric variations using light sources and photodetectors. Oximetry, used for SpO_2_ measurement, operates on a similar principle but requires two light sources of different wavelengths [37]. RR can be deduced from pulse wave modulation [38,39] or using motion sensors [40,41]. Therefore, a severity score system based on these three parameters appears relevant. Examples of a lab-built prototype and an already commercialised wearable device are given in S1 Fig.

This paper introduces a new trauma severity score, S_HRO_, tailored for wearable monitoring and calculated from three intermediate score functions: S_H_, S_R_, and S_O_, respectively corresponding to heart rate, respiratory rate, and SpO_2_. These functions are developed using a sigmoid model, and boundary conditions are defined through a survey among expert health professionals. The intermediate scores are then combined using a specially designed logical function, allowing for the consideration of more than three parameters. Thus, the new scoring system has been designed based on theoretical considerations and expert input, rather than through a data-driven approach, such as one based on machine learning. It has been tested on a database of subjects admitted to the intensive care unit by comparing the classification made with the new score and the independent evaluation by physicians and medical staff considered as a reference. In addition, two examples of continuous monitoring over time are given. The paper also discusses several options for improving this initial version of the new scoring system.

## Materials and methods

### A new severity scoring system calculated from heart rate, respiration rate, and SpO_2_

#### General score conception.

The physiological parameters HR, RR, and SpO_2_ are respectively denoted as x_H_, x_R_, and x_O_ serving as function antecedents. The comprehensive score S_HRO_(x_H_, x_R_, x_O_) is derived from three intermediate scores corresponding to each vital sign: the heart rate score S_H_(x_H_), the respiratory rate score S_R_(x_R_), and the SpO_2_ score S_O_(x_O_). These scores, ranging from 0 to 100, adhere to the principle that a higher score indicates a more severe medical condition. Additionally, the scores are colour-coded similarly to the Simple Triage And Rapid Treatment (START) protocol algorithm: green for low-priority casualties, orange for delayable ones, red for urgent cases, and black for deceased or unexpectant individuals [42–44]. Consequently, the score intervals [0,33], [33,67], and [67,100] correspond respectively to the colours green, orange, and red.

**Fig 1:**
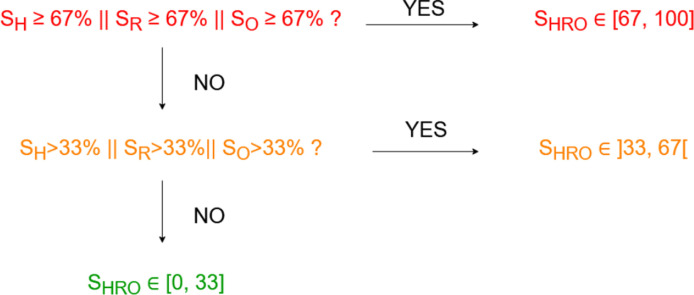
Logical tree representing the rules used for determining the global score S_HRO_’s range. The latter depends on the ranges of the heart score S_H_, the respiratory score S_R_, and the SpO_2_ score S_O_.

For heart and respiratory rates, normal values fall within a specific range, with deviations from this range indicating increasing criticality. Therefore, the functions S_H_(x_H_) and S_R_(x_R_) exhibit two segments: one decreasing and the other increasing. In contrast, a normal SpO_2_ value is close to the maximum (100%), making the S_O_(x_O_) score function a monotonically decreasing one. These intermediate scores S_H_, S_R_, and S_O_ are modelled using sigmoid functions. A survey was conducted to establish normal, abnormal, and critical values for the three physiological parameters, each associated with the above-mentioned colour codes green, orange, and red. Furthermore, a specialized function f_merge_ has been devised to amalgamate the three intermediate scores into the overarching score S_HRO_, which aims to reflect the overall health status. In the realm of health scoring, the merging function adheres to specific logical rules: if any intermediate score is red, the global score is red; if none are red but at least one is orange, the global score is orange; and the global score is green only if all intermediate scores are green. These logical rules are illustrated in Fig 1. The development processes of the intermediate scores and of the merging function are described in the following paragraphs.

#### Intermediate scores’ boundary values.

**Fig 2:**
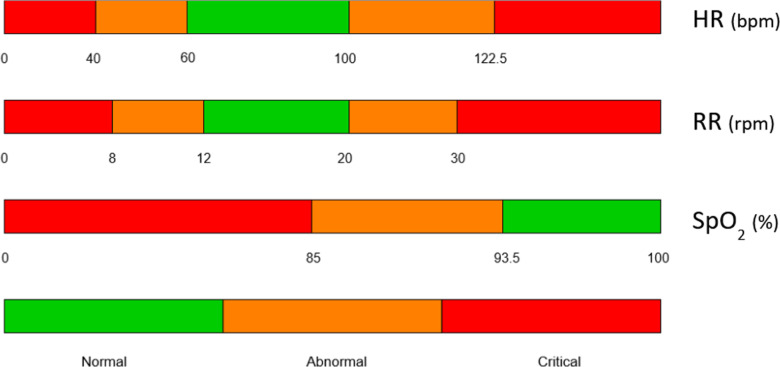
Boundaries of normal, abnormal, and critical value ranges for heart rate (HR), respiratory rate (RR), and blood oxygenation (SpO_2_).

Normal, abnormal, and critical values for the three physiological parameters are respectively represented by scores within the intervals [0,33] (green), [33,67] (orange), and [67,100] (red). For each vital sign, the boundary values x_P,b_ correspond to the points at which the score colour changes. These are the values whose images, as determined by the intermediate score functions S_P_(x_P_) where P ∈  {H, R, O}, equal 33 or 67 (as shown in Equation 1).


$$x_{P,b}=\left\{\left.x\right|S_{P}\left(x_{P}\right)\epsilon \left\{33,67\right\}\right\},\text{P}\epsilon \left\{\text{H},\text{R},\text{O}\right\}$$ (1)

where, S_P_ represents the score function for the physiological parameter x_P_. The Modified Early Warning Score coding system provides a basis for understanding the normal, abnormal, and critical values of heart rate, respiratory rate, and SpO_2_ [45]. However, MEWS employs four levels of severity, whereas our approach requires only three. Due to the absence of suitable boundary values in existing literature, a survey was conducted among expert health professionals to establish these values. The survey participants, drawn from the French Society of Anesthesia and Intensive Care (Société Française d’Anesthésie et de Réanimation), were asked to identify boundary values for an adult subject for each of the three physiological parameters. These values are illustrated in Fig 2. Details can be found in S1 File.

#### Sigmoid model’s parameters of the intermediate score functions.

The intermediate score functions S_H_(x_H_), S_R_(x_R_), and S_O_(x_O_) are modelled using sigmoid functions. Since S_O_(x_O_) is monotonic, a single sigmoid term suffices (Equation 2).


$$S_{O}\left(x\right)=\frac{100}{1+e^{a_{1}x+b_{1}}}$$ (2)

S_H_(x_H_) and S_R_(x_R_) functions display two segments with differing directional variations. Consequently, they are modelled by a superposition of two sigmoid functions (Equation 3).


$$S_{P}\left(x\right)=\frac{100}{1+e^{a_{1}x+b_{1}}}+\frac{100}{1+e^{a_{2}x+b_{2}}}\text{for P}\epsilon \left\{\text{H},\text{R}\right\}$$ (3)

where a_1_, a_2_, b_1_, b_2_ correspond to the sigmoid functions’ parameters.

For each vital sign, the parameters a_1_, a_2_, b_1_, b_2_ must be determined. Based on the survey conducted among expert health professionals, boundary values delimiting normal, abnormal, and critical ranges have been established. These values serve as boundary conditions, as specified in Equation 4.


$$\{\begin{array}{c} S_{O}\left(x_{O}\_1\right)=33\\ S_{O}\left(x_{O}\_2\right)=67 \end{array}$$

$$\{\begin{array}{c} S_{P}\left(x_{P}\_1\right)=67\\ S_{P}\left(x_{P}\_2\right)=33\\ S_{p}\left(x_{P}\_3\right)=33\\ S_{p}\left(x_{P}\_4\right)=67 \end{array}\text{for P }\epsilon \left\{\text{H},\text{R}\right\}{\text{and x}}_{\text{P}}\epsilon \left\{\text{HR},\text{RR}\right\}$$ (4)

**Fig 3:**
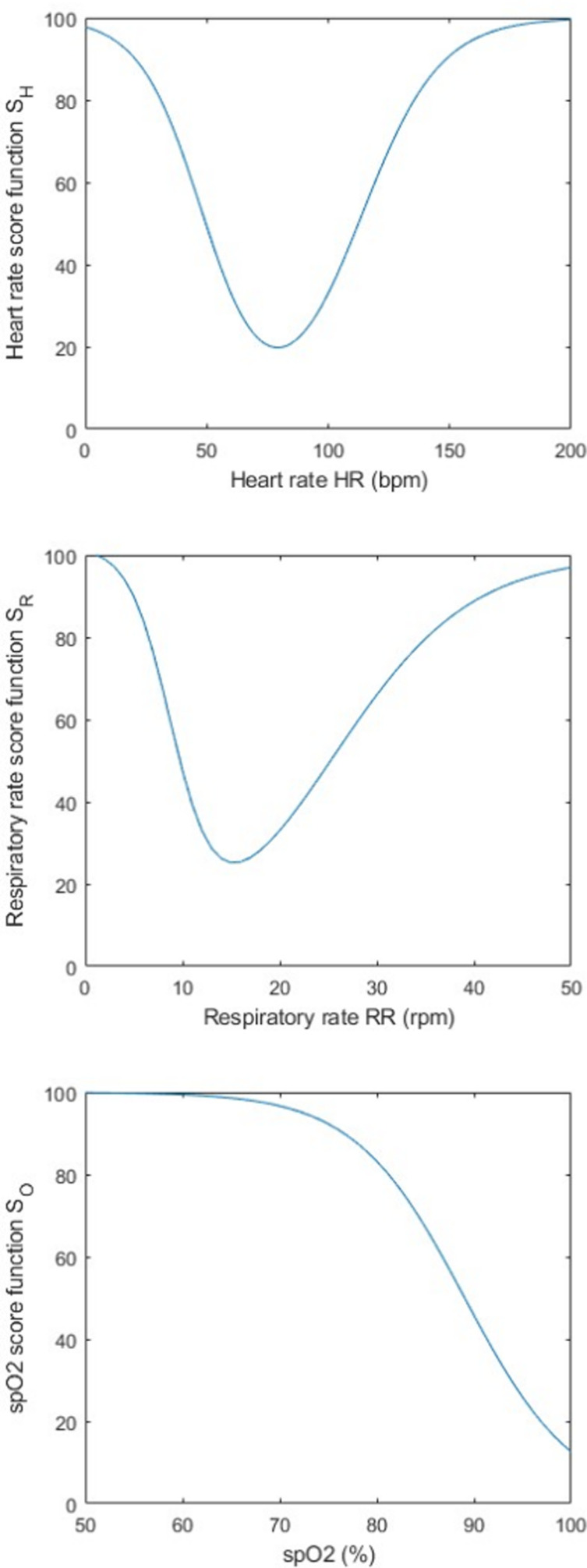
Intermediate score functions of heart rate S_H_(x_H_), respiratory rate S_R_(x_R_), and SpO_2_ S_O_(x_O_) obtained using a sigmoid model. The boundary conditions have been defined thanks to the survey conducted among expert health professionals.

The system was resolved using curve-fitting techniques. The resulting parameters a and b are presented in Table 1. For each physiological parameter, the coefficient of determination R2 equals 1, indicating a successful resolution of the system through curve-fitting. The resulting intermediate score functions S_H_(x_H_), S_R_(x_R_), and S_O_(x_O_) are illustrated in Fig 3.

**Table 1:** Parameters a and b computed following a sigmoid model for the intermediate score functions of heart rate S_H_(x_H_), respiratory rate S_R_(x_R_), and SpO_2_ S_O_(x_O_).

Function	a_1_	b_1_	a_2_	b_2_	R2	SSE
S_H_(x_H_)	0.07701	-3.738	-0.06142	6.936	1	6.058e^-28^
S_R_(x_R_)	0.4431	-3.903	0.1403	3.545	1	1.998e^-21^
S_O_(x_O_)	0.177	-15.76			1	3.175e^-18^

The coefficients of determination (R2) and the sums of square error (SSE) are also given.

#### Merging function.

The global score S_HRO_(x_H_,x_R_,x_O_) is computed using a merging function f_merge_ based on the three intermediate scores S_H_, S_R_, and S_O_, derived from the previously developed functions (Equation 5).


$$S_{HRO}=f_{merge}\left(S_{H}\left(x_{H}\right),S_{R}\left(x_{R}\right),S_{O}\left(x_{O}\right)\right)$$ (5)

The function f_merge_ is presumed to be symmetric and must satisfy two conditions: (i) adherence to the logical tree depicted in Fig 1, and (ii) evolutionary behaviour. Condition (ii) implies that f_merge_ should reflect the cumulative effect of the intermediate scores: if S_1_ < S_2_, then f_merge_(S_1_, S_X_, S_Y_) < f_merge_(S_2_, S_X_, S_Y_). For instance, a subject with an orange S_H_ value and green S_R_ and S_O_ values should have a S_HRO_ score lower than a subject with three orange intermediate scores. Without fulfilling these conditions the maximum max (S_H_, S_R_, S_O_) and average mean (S_H_, S_R_, S_O_) could have been used as the merging function; however, they respectively do not meet conditions (i) and (ii).

A code array is introduced, containing an element for each physiological parameter. Each code element is assigned a value of 0, 1, or 2, corresponding to the score intervals [0;33] (green), [33;67] (orange), or [67;100] (red), as defined in Equation 6.


$$\left[C_{H},C_{R},C_{O}\right]with |\begin{array}{c} C_{P}=0if0\leq S_{P}\leq 33\\ C_{P}=1if33<S_{P}<67\\ C_{P}=2if67\leq S_{P}\leq 100 \end{array}P=H,R,O$$ (6)

Given the symmetry of f_merge_, the code array can be viewed as a 3-combination with repetition {C_H_, C_R_, C_O_} from a set {0, 1, 2}. If n vital signs were to be used in the global score calculation instead of 3, this could be generalized to an n-combination with repetition {C_1_, C_2_, … C_n_} from the set {0, 1, 2}.

The output range of the merging function is determined by the maximum element in the code array, in accordance with condition (i): it is red if the maximum is 2, orange if it is 1, and green if it is 0 (refer to Table 2, column “Maximum”). Additionally, the sum of the code array elements helps to determine the appropriate subinterval, in line with condition (ii). This sum represents a weighted count of the green, orange, and red intermediate scores. The range of this sum is specified in Table 3 (columns “sum_MIN_” and “sum_MAX_”) for each interval, leading to the calculation of the number of possible subintervals N_SI_ as per Equation 7 (Table 3, column “N_SI_”). S2 File provides mathematical details on the number of combinations that result in green, orange, or red outputs, in relation to Fig 1.

**Table 2:** Relationship between the merging function’s output interval and the input code array’s maximum, sum range (sum_MIN_ and sum_MAX_), and the number of possible subintervals N_SI_ for n physiological parameters.

Interval	Colour	Maximum	sum_MIN_	sum_MAX_	N_SI_
[0;33]	Green	0	0		1
[33;67]	Orange	1	1	n	n
[67;100]	Red	2	2	2n	2n-1

**Table 3:** Merging function’s subintervals lower and upper bounds in the case of three physiological parameters (n = 3).

Combination	Max	Sum	subInt_Low	subInt_up
{0,0,0}	0	0	0	33
{0,0,1}	1	1	33	44,3
{0,1,1}		2	44,3	55,7
{1,1,1}		3	55,7	67
{0,0,2}	2	2	67	73,6
{0,1,2}		3	73,6	80,2
{0,2,2}, {1,1,2}		4	80,2	86,8
{1,2,2}		5	86,8	93,4
{2,2,2}		6	93,4	100


$$N_{SI}=sum_{MAX}-sum_{MIN}+1$$ (7)

The intervals are then evenly divided into the calculated number of subintervals. The lower and upper bounds of these subintervals are determined as outlined in Equation 8.


$$subInt_{LOW}=int_{LOW}+\frac{int_{UPP}-int_{LOW}}{N_{SI}}*\left(sum\left(codeArray\right)-sum_{MIN}\right)$$

$$subInt_{UPP}=int_{LOW}+\frac{int_{UPP}-int_{LOW}}{N_{SI}}*\left(sum\left(codeArray\right)-sum_{MIN}+1\right)$$ (8)

For instance, with n = 3 physiological parameters, Table 3 indicates that there is 1 possible green subinterval (N_SI_ = 1), 3 possible orange subintervals (N_SI_ = n = 3), and 5 possible red subintervals (N_SI_ = 2n-1 = 5). The lower and upper bounds of these subintervals, as calculated using Equation 8, are detailed in Table 3.

The merging function has been defined with Equation 9:


$$f_{merge}\left({\left\{S_{i}\right\}}_{1\leq i\leq n}\right)=subInt_{LOW}$$

$$+\frac{mean\left({\left\{S_{i}\right\}}_{1\leq i\leq n}\right)-mean\left({\left\{min_{i}\right\}}_{1\leq i\leq n}\right)}{mean\left({\left\{max_{i}\right\}}_{1\leq i\leq n}\right)-mean\left({\left\{min_{i}\right\}}_{1\leq i\leq n}\right)}$$

$$*\left(subInt_{UPP}-subInt_{LOW}\right)$$

$$where\;\begin{array}{c} min_{i}=0if0\leq S_{i}\leq 33\\ min_{i}=33if33<S_{i}<67\\ min_{i}=67if67\leq S_{i}\leq 100 \end{array}\;and$$

$$\begin{array}{c} max_{i}=33if0\leq S_{i}\leq 33\\ max_{i}=67if33<S_{i}<67\\ max_{i}=100if67\leq S_{i}\leq 100 \end{array}$$ (9)

**Fig 4:**
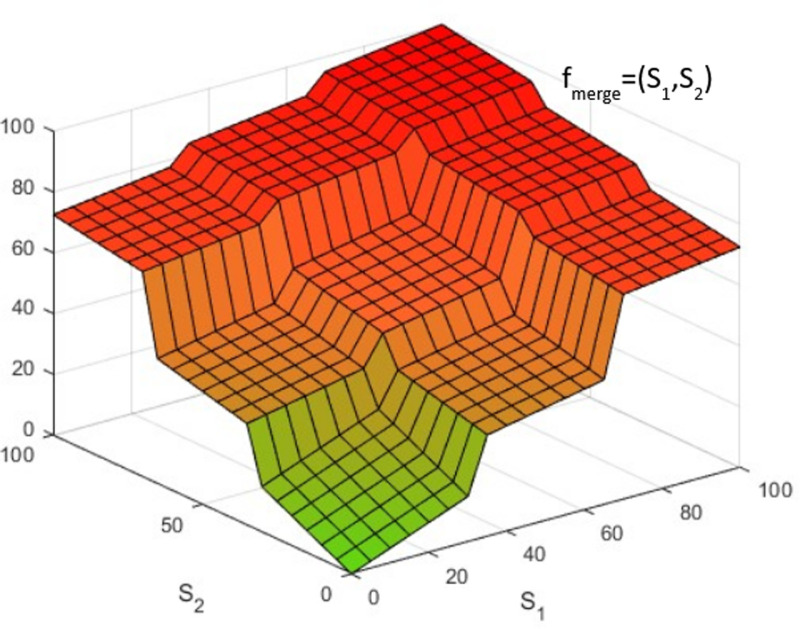
3D plot of f_merge_ in the case of two input physiological parameters S_1_ and S_2_ (n = 2).

Fig 4 presents a 3D plot of f_merge_ in the scenario of two input physiological parameters (n = 2).

In summary, this new trauma severity scoring system, tailored for wearable monitoring, operates in two stages. Initially, intermediate scores for heart rate, respiratory rate, and SpO_2_ are calculated using the functions S_H_(x_H_), S_R_(x_R_), and S_O_(x_O_). Subsequently, the global score S_HRO_ is computed using the merging function f_merge_(S_H_, S_R_, S_O_), as elaborated above. This global score can be continuously computed from the three physiological parameters measured by a wearable device, eliminating the need for manual evaluation by a health professional for the score calculation.

#### Database’s constitution.

To evaluate the effectiveness of the newly developed scoring system, it was tested with real-life data. We used data from major patients admitted for trauma to the Intensive Care Unit (ICU) of La Timone Teaching Hospital in Marseille and included in the project PHYSIOS (RO-2015/17, reference 2015-33). This project was approved by the Personal Protection Committee 1 South Mediterranean (*Comité de Protection des Personnes Sud Méditerranée I*). According to French regulations, the patients were informed in writing that their anonymous data would be used for research purposes and that they could opt-out anytime. (As this was an observational study and data were anonymously analysed, written consent was not required.) The assessment period spanned from 1 May 2022 to 30 June 2023. The database included the heart rate (HR), respiratory rate (RR), and peripheral oxygen saturation (SpO_2_) of patients admitted to the trauma room. The measurements were carried out with the hospital’s scope devices, and the variables consisted of arrays of these physiological measures over time.

Using the intermediate score functions, the intermediate scores S_H_, S_R_, and S_O_ were respectively calculated from the HR, RR, and SpO_2_ arrays. To synchronize the time arrays of these three scores, an interpolation process was applied. Subsequently, a global score array for each subject was computed using the f_merge_ function. Additionally, physicians conducted separately a colour code evaluation (green, orange, or red) for each subject, providing a clinical perspective on the severity of their condition. They were completely blinded to all the information provided by the model.

This assessment aims to validate the scoring system’s utility in a real-world clinical setting, particularly its potential for continuous patient monitoring and its accuracy in reflecting patient conditions as evaluated by medical professionals.

## Results

### Classification

#### Database description.

The dataset was found to be complete for 84 subjects, categorized as 19 green, 37 orange, and 28 red (or black) based on the independent physicians’ evaluations. Notably, 22 subjects had also neurological lesions. Both intermediate and global scores were averaged over time for analysis. For 17 subjects, only initial time points (prior to any care or sedation) were considered to accurately represent the subject’s health status at admission.

**Fig 5:**
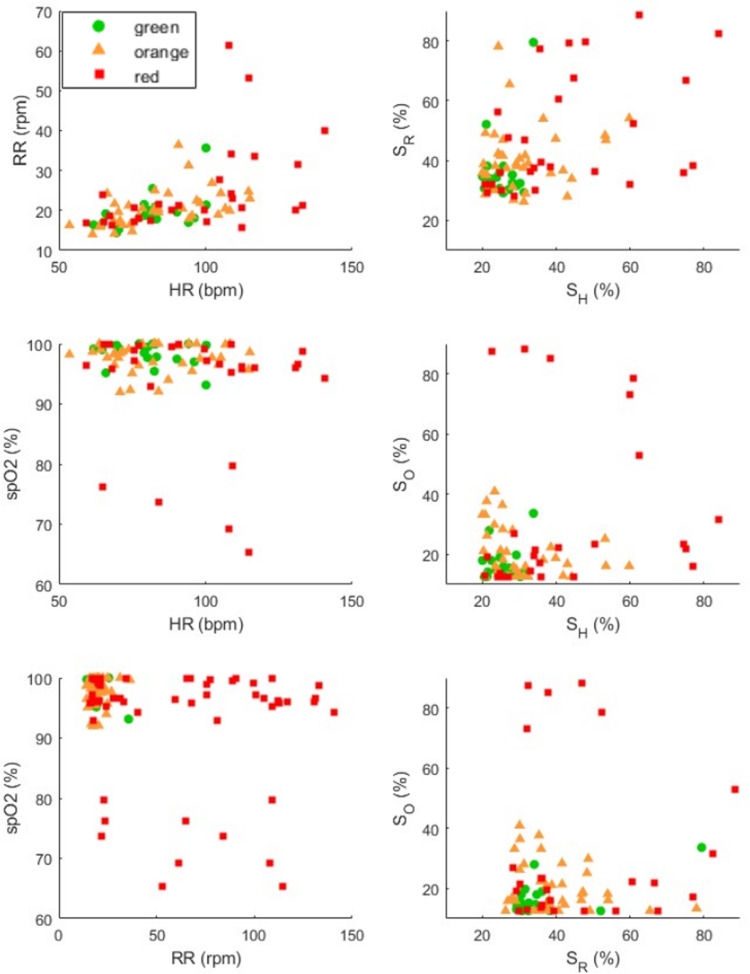
Repartition of the constituted database subjects’ heart rate (HR), respiratory rate (RR) and SpO_2_, as well as intermediate scores S_H_, S_R_ and S_O_ respectively associated to the physiological parameters.

The distribution of the constituted database subjects’ physiological parameters is displayed in Fig 5, on the left column. It appears that the subjects exhibit various values of heart rate, respiratory rate, and SpO_2_. The database’s diversity ensures that the prediction success rates calculated in this part are significant. The equivalent display is given on the right column using the intermediate scores. No distinct clusters of green, orange, and red subjects appear clearly, even if it can be noticed that the subjects with high intermediate scores are mostly red. Correlations between the intermediate scores have been checked. Heart and respiratory rates seem to be slightly correlated (0.49), while SpO_2_ is decorrelated from both HR and RR (respectively 0.24 and 0.28).

**Fig 6:**
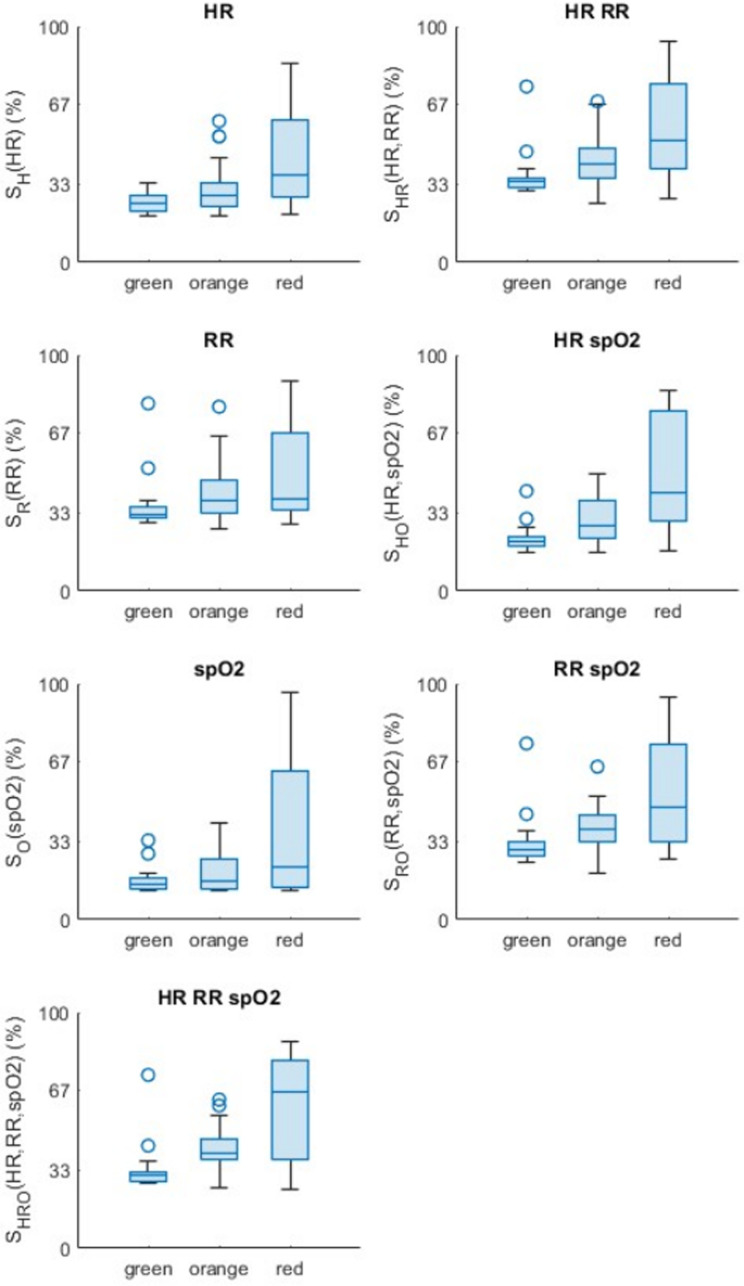
Boxplots of score computed with 1,2 or 3 physiological parameters (among HR, RR and SpO_2_) represented for each group of subjects evaluated as green, orange or red by physicians.

Boxplots of the intermediate scores S_H_, S_R_, and S_O_, the scores calculated with two of the three physiological parameters S_HR_, S_HO_, and S_RO_, and the global score S_HRO_ are given in Fig 6. For all scores, the median values tend to increase as the colour goes from green to red (even if for S_R_, the median scores are equivalent between orange and red subjects; as well as for S_O_ between green and orange subjects). Therefore, the gravity scores that have been developed seem to be consistent. It also appears on all graphs that the score range is limited for green subjects, is higher for orange ones, and even more for the reds. Moreover, one can note that the median values seem to differ more between the three colour groups when increasing the number of physiological parameters considered in the score.

#### Assessment of the scoring system’s prediction success.

In order to assess the new scoring system, the colour classification made with the scores (green for [0,33], orange for [33,67], red for [67,100]) has been compared with the independent colour evaluation carried out by physicians. The prediction success rates (PSR) have been calculated as the ratio of correctly predicted subjects Ns_correct_ over the total number of subjects Ns_tot_. The prediction success rates for the different scores using 1, 2, or 3 physiological parameters are given in Table 4.

**Table 4:** Prediction success rates using 1, 2, or 3 physiological parameters (among heart rate, respiratory rate and SpO_2_) across the 62 non-neurological subjects, the 22 neurological subjects, and the entire database.

Considered parameters			Prediction success rate		
HR	RR	SpO_2_	Non-neurological (62)	Neurological (22)	All (84)
x			47%	14%	38%
	x		57%	41%	52%
		x	39%	27%	36%
x	x		57%	59%	57%
x		x	58%	36%	52%
	x	x	68%	50%	63%
x	x	x	77%	64%	74%


$$PSR=\frac{Ns_{correct}}{Ns_{tot}}*100$$ (10)

The prediction success rates have been calculated considering only the non-neurological subjects, only the neurological ones, and on the entire database.

**Fig 7:**
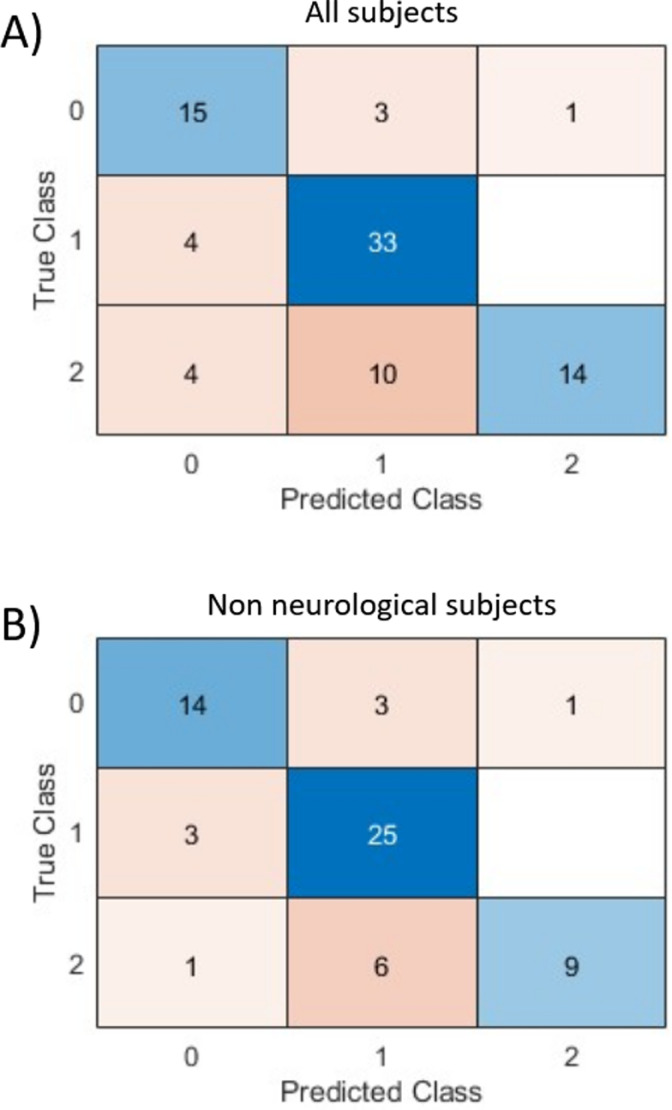
Confusion matrix of colour code prediction using the new scoring system compared to the colour evaluation carried out by physicians as the true class for all subject (A) and for non-neurological subjects (B). 0: green, 1: orange, 2: red.

The prediction success rate of the global score over the entire database is 74%: 62 of 84 subjects have been correctly predicted by the new scoring system. The confusion matrix is given in Fig 7A. Among the 22 incorrectly predicted subjects, 8 are neurological subjects, two of them are alcohol-intoxicated, and 6 exhibit a global score value close to the value range it should have belonged to (among which 2 are also either neurological or alcohol-intoxicated). Without considering the neurological subjects, a prediction success rate of 77% is even achieved. The corresponding confusion matrix is given in Fig 7B.

**Fig 8:**
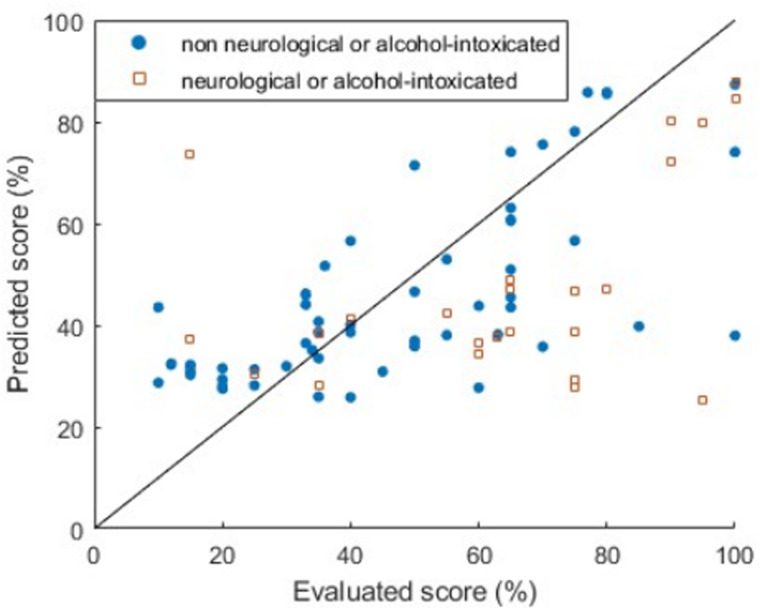
Calibration curve: predicted scores using S_HRO_ as a function of the scores evaluated by physicians.

Model calibration has also been checked; the calibration curve is presented in Fig 8. The Expected Calibration Error (ECE) has been calculated using a uniform binning with 5 bins: it equals 0.28. While the threshold values 33 and 67 have been used to classify the subjects according to the colour code, optimised thresholds have been defined in the S3 File. However, it has not led to better prediction success rate.

#### Continuous monitoring.

**Fig 9:**
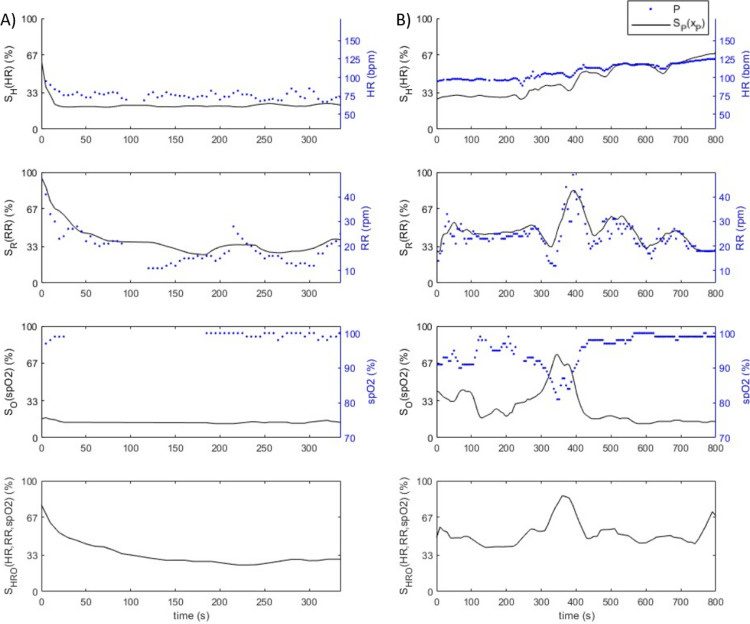
Evolution over time of two subjects A and B’s heart rate, respiratory rate, SpO_2_ and the associated intermediate scores S_H_(HR), S_R_(RR), S_O_(SpO_2_), as well as of the global score S_HRO_(HR, RR, SpO_2_). The physiological parameters P and the scores S_P_(x_P_) are represented respectively with blue dots and continuous black line. The scores S_H_, S_R_, S_O_ and S_HRO_ have been smoothed over the equivalent of respectively 30, 60, 30 and 30 seconds.

The advantage of the new trauma severity score is to enable health professionals to continuously monitor patients, even outside the ICU or the hospitals. Two examples are given in Fig 9. Heart rate, respiratory rate, and SpO_2_ are plotted over a few minutes on the right axis of the three upper graphs (blue dots). The intermediate scores associated with the three physiological parameters are also displayed on the left axis (continuous black lines). Finally, on the lower graph, the evolution of the global score over time is given.

In Fig 9A, the heart rate decreases quickly (in less than 30 seconds), while the respiratory rate decreases over a longer period of 2.5 minutes. Therefore, the intermediate scores S_H_ and S_R_ decrease as well (respectively from 63% to around 20% and from 96% to around 25%). SpO_2_ is consistently very close to 100%; S_O_ is consequently more or less constant and low (15%). Consistent with S_H_ and S_R_, the global score decreases from 78% (red) to around 33% (orange/green). This decrease reflects an improvement in the medical status of subject A, which is related to the care received when managed in the ICU.

In Fig 9B, the heart rate intermediate score increases from 27% (green) to 68% (red), as the heart rate increases from 95 bpm to 125 bpm during the 13 minutes of monitoring. The SpO_2_ value is above 90% during the first 5 minutes and then decreases to reach a critical value of 81% (at around 350 seconds) after the beginning of the monitoring. Thus, the SpO_2_ score S_O_ varies from around 33% (green/orange) to 81% (red). At the same moment, the respiratory rate accelerates, which could be related to this SpO_2_ decrease. From 4.5 minutes, the respiratory rate and SpO_2_ stabilize: S_R_ remains in orange, while S_O_ returns to green values. These different elements result in a global score fluctuating between orange and red values.

These two examples illustrate how the new scores can be interpreted and used as a tool by health professionals to follow patients’ health status evolution over time. Peterson describes a recommendation of monitoring frequency depending on the EWS value [15]. With the new severity scoring system, this question no longer arises since the score calculation is made directly from HR, RR, and SpO_2_, without requiring a professional evaluation. The frequency of score computation should therefore be determined based on the system’s processing capacity and data management to avoid latency issues and to ensure an optimal balance of collected data. This tool can be especially useful in the case of patient monitoring to detect in real-time deterioration or to quantify stability.

## Discussion

The obtained prediction success rates when comparing the colour classification made with the scores and the independent colour evaluation conducted by physicians (Table 4), for instance 74% over the entire database when considering HR, RR and SpO_2_, show that the new trauma scoring system is relevant. Notably, the system’s efficacy is particularly pronounced in cases involving non-neurological or non-alcohol-intoxicated subjects, where the PSR rises to 77% upon excluding these individuals compared to 64% when considering only them. Even though a prediction success rate of 74% is statistically significant, it does not meet the rigorous demands of clinical practice, where the stakes involve human lives. Nevertheless, it must be noted that the four subjects of the database that died presented a global score higher than 74%. It suggests a potential correlation between higher scores and more severe outcomes, which requires further investigation.

Since it can be noted in Table 4 that the prediction success rate is improved when increasing the number of physiological parameters, the scoring system may be upgraded by introducing other physiological parameters allowing the evaluation of neurological aspects, what is easily possible given the way the merging function of the intermediate score has been designed. In the case of non-hospitalized individuals, motion is an easily measurable parameter with accelerometers and gyroscopes. Typically, an injured individual who is immobile is immediately considered to be in a severe condition. Besides the motion level, data processing can include walk and fall detection. If the wearable included a user interface (screen, or microphone and speakers), a consciousness level test evaluation could also be conceived, as a substitute for the AVPU and GSC scores. Moreover, Electrodermal Activity (EDA) and Heart Rate Variability (HRV) can be used to quantify the sympathetic/parasympathetic balance of the autonomous nervous system in relation to stress and pain [24,46–49]. These development paths should especially be effective for neurological subjects. Depending on the application, a haemorrhagic risk function could also be developed from pulse wave amplitude and heart rate [50,51]. The intermediate score functions may additionally be integrated in a more complex algorithm considering a broader array of information. Depending on the application scenario, the available inputs, such as age, body composition, medical history, or ongoing treatment (medication administration, oxygen assistance, etc.), may vary. Furthermore, the new scoring systems seem to rather underestimate the colour class according to Fig 7, while it is rather desirable to overestimate the gravity to avoid medical mistakes in the case of mass casualty situations. However, the results presented here only represent a first attempt and several optimisations to improve the predictive capability have been given above.

It is also interesting to analyse which physiological parameters seem to have the best predictive capability. Among the three intermediate scores, e.g., when considering either HR, RR, or SpO_2_ (Table 5), the prediction success rate is higher for respiratory rate (57% versus 47% for HR and 39% for SpO_2_). This is also reflected in the scores using two physiological parameters since S_HR_ and S_RO_‘s prediction success rates are higher than S_HO_’s one. It can be even noticed that the predictive success rate when using only RR is equivalent to the one when using both HR and SpO_2_. Nevertheless, when considering the scores computed with optimised thresholds in S3 File, the PSRs are equivalent to each other when considering one vital sign (between 49 and 54%). Thus, HR, RR and SpO_2_ seem to exhibit a similar predictive weight if the colour limit thresholds are optimised, even though it can be noticed on the ROC curves on S3 File that the areas under curves are slightly higher for HR (above 0.72) than for RR (0.65; 0.69) and for SpO_2_ (0.64; 0.66). The thresholds without optimisation were somehow more or less adapted for respiration rate. An additional study should also be carried out to identify which of the neurological physiological parameters mentioned above are the most efficient.

In this study, the new trauma severity scoring system has been assessed using a database only including patients admitted to the intensive care unit, what may affect the mobility of the patients and the natural progression of their conditions. All patients had undergone some form of medical intervention before and during their assessment, which could influence the model’s predictive accuracy. Therefore, this pilot study should be completed by testing the new trauma score test on additional multi-centric datasets including both individuals with compromised health and healthy subjects. Moreover, the measurements have been carried out using hospital’s scope devices, whereas the scoring system aims to be combined with wearable technologies. Further investigation should consequently be carried out to identify suitable medical-grade monitoring wearables, check their measurement reliability in real prehospital conditions and its influence on the computed scores, and integrate intermediate functions into a more complex algorithm taking account of additional inputs.

## Conclusions

A novel trauma scoring system tailored for wearable monitoring has been developed, deriving calculations from three key physiological parameters: heart rate (HR), respiratory rate (RR), and peripheral oxygen saturation (SpO_2_). Medical-grade devices enabling precise measurement of these physiological parameters should become increasingly available as challenges related to wearables technologies (such as ergonomics, data consistency, etc.) are addressed. Intermediate scores S_H_, S_R_, and S_O_ are initially computed from these vital signs using a sigmoid model, with boundary conditions established through a survey among French health professionals (54 responses). Additionally, a merging function f_merge_ has been introduced to amalgamate these intermediate scores, offering flexibility for incorporating more than three parameters in future enhancements of the system.

The efficacy of the system was tested using a database of 84 subjects from an intensive care unit. The scoring system’s colour codes were validated against physicians’ independent assessments, achieving a prediction success rate of 74% across the entire database and the Expected Calibration Error equals 0.28. This underscores the relevance of the chosen physiological parameters and incorporating additional parameters could further refine the prediction accuracy. Two case studies demonstrated the utility of this new monitoring score for continuous health status tracking. As the score is automatically calculated from wearable measurements, bypassing the need for human evaluation, monitoring frequency is no longer a constraint, provided sufficient computational resources are available. This score serves as the initial development phase of a real-time multi-patient monitoring tool that enable the detection of sudden deterioration in a patient’s condition. This tool’s capability to detect sudden patient condition deteriorations could be invaluable in MCIs, assisting in the optimal allocation of medical resources and enhancing patient care.

While HR, RR, and SpO_2_ effectively represent an individual’s hemodynamic and respiratory status, the current version of the scoring system lacks a neurological dimension. To address this, integrating additional physiological parameters such as motion, electrodermal activity, or heart rate variability is proposed, potentially enhancing the accuracy, particularly for neurological cases. An additional study including all available physiological parameters could explore which ones are the most relevant and identify the redundant ones, for instance by carrying out a principal component analysis. Optimising intermediate scores also represents an improvement target. This study is set against the backdrop of ongoing advancements in hardware technologies and data processing, suggesting that the proposed severity scoring system is poised for continuous evolution.

Despite its potential for real-time health monitoring, the current system does not account for historical health data. Introducing thresholds that prevent the score from dropping below certain levels after exceeding set limits could be beneficial. Additionally, the quality of vital sign measurements by the wearable device warrants consideration. Exploring fuzzy logic as an alternative to the f_merge_ function could offer a more nuanced approach to integrating intermediate scores [16]. Expanding the database and employing machine learning algorithms could further refine the system. For everyday wearables, personalizing intermediate score functions based on user-specific factors like age could significantly enhance the system’s precision. In such scenario, detecting abnormal vital signs, falls, or accidents could automatically trigger communication with emergency services.

In conclusion, this initial version of the wearable monitoring-adapted scoring system is shows promise for integration in a more complex algorithm including additional inputs and accounts for the measurement quality of the wearable devices (sensitivity, reliability, selectivity, etc.). Further validation is required with a broader range of data, encompassing both individuals with compromised health and healthy subjects. Moreover, this score is aimed to be continuously updated in line with technological advancements and integrating additional physiological parameters as they become clinically validated for use in wearables.

## Supporting information

S1 Fig A) Arduino wearable prototype integrating a Wio Terminal (acceleration) and a Maxim integrated PPG/oximetry/temperature sensor; B) EmbracePlus smartwatch (Empatica) enabling EDA, PPG, acceleration and temperature monitoring. (TIF)

S1 File Survey conducted to determine intermediate scores’ boundary values. (DOCX)

S2 File Merging function: class’ number of combinations. (DOCX)

S3 File Colour limit thresholds’ optimisation. (DOCX)

## References

[1] Kim D, Lee D, Lee B, Cho Y, Ryu S, Jung Y. Performance of modified early warning score (MEWS) for predicting in-hospital mortality in traumatic brain injury patients. J Clin Med. 2021;10(9):1915. 33925023 10.3390/jcm10091915 PMC8124302

[2] Kondo Y, Abe T, Kohshi K, Tokuda Y, Cook EF, Kukita I. Revised trauma scoring system to predict in-hospital mortality in the emergency department: Glasgow Coma Scale, age, and systolic blood pressure score. Crit Care. 2011;15(4):R191. doi: 10.1186/cc10348 21831280 PMC3387633

[3] Jeong JH, Park YJ, Kim DH, Kim TY, Kang C, Lee SH, et al. The new trauma score (NTS): a modification of the revised trauma score for better trauma mortality prediction. BMC Surg. 2017;17(1):77. doi: 10.1186/s12893-017-0272-4 28673278 PMC5496419

[4] Heydari F, Azizkhani R, Ahmadi O, Majidinejad S, Nasr-Esfahani M, Ahmadi A. Physiologic scoring systems versus Glasgow Coma Scale in predicting in-hospital mortality of trauma patients; a diagnostic accuracy study. Arch Acad Emerg Med. 2021;9(1):e64. doi: 10.22037/aaem.v9i1.1376 34870230 PMC8628642

[5] Khari S, Zandi M, Yousefifard M. Glasgow Coma Scale versus physiologic scoring systems in predicting the outcome of ICU admitted trauma patients; a diagnostic accuracy study. Arch Acad Emerg Med. 2022;10(1):e25. 35573721 10.22037/aaem.v10i1.1483 PMC9078058

[6] Ying Y, Huang B, Zhu Y, Jiang X, Dong J, Ding Y, et al. Comparison of five triage tools for identifying mortality risk and injury severity of multiple trauma patients admitted to the emergency department in the daytime and nighttime: a retrospective study. Appl Bionics Biomech. 2022;2022:9368920. doi: 10.1155/2022/9368920 35251304 PMC8896924

[7] Durantez-Fernández C, Martín-Conty J, Medina-Lozano E, Mohedano-Moriano A, Polonio-López B, Maestre-Miquel C. Early detection of intensive care needs and mortality risk by use of five early warning scores in patients with traumatic injuries: an observational study. Intensive Crit Care Nurs. 2021;67:103095. 34244029 10.1016/j.iccn.2021.103095

[8] Yu Z, Xu F, Chen D. Predictive value of Modified Early Warning Score (MEWS) and Revised Trauma Score (RTS) for the short-term prognosis of emergency trauma patients: a retrospective study. BMJ Open. 2021;11(3):e041882. doi: 10.1136/bmjopen-2020-041882 33722865 PMC7959230

[9] Galvagno SM, Massey M, Bouzat P, Vesselinov R, Levy MJ, Millin MG, et al. Correlation between the revised trauma score and injury severity score: implications for prehospital trauma triage. Prehospital Emergency Care. 2019;23(2):263–70. 30118369 10.1080/10903127.2018.1489019

[10] Vivien B, Riou B, Carli P. Critères et scores de gravité. Urgences; 2008.

[11] Fani-Salek M, Totten V, Terezakis S. Trauma scoring systems explained. Emerg Med. 1999;11(3):155–66.

[12] Sternbach G. The Glasgow Coma Scale. J Emerg Med. 2000;19(1):67–71. doi: 10.1016/S0736-4679(00)00112-0 10863122

[13] Barlow P. A practical review of the Glasgow Coma Scale and Score. Surgeon. 2012;10(2):114–9. doi: 10.1016/j.surge.2011.12.003 22300893

[14] Fullerton J, Price C, Silvey N, Brace S, Perkins G. Is the Modified Early Warning Score (MEWS) superior to clinician judgement in detecting critical illness in the pre-hospital environment? Resuscitation. 2012;83(5):557–62. 22248688 10.1016/j.resuscitation.2012.01.004

[15] Petersen JA. Early warning score challenges and opportunities in the care of deteriorating patients. Dan Med J. 2018;65(2):B5439. 29393044

[16] Al-Dmour JA, Sagahyroon A, Al-Ali AR, Abusnana S. A fuzzy logic-based warning system for patients classification. Health Informatics J. 2019;25(3):1004–24. doi: 10.1177/1460458217735674 29108458

[17] Cooksley T, Kitlowski E, Haji-Michael P. Effectiveness of Modified Early Warning Score in predicting outcomes in oncology patients. QJM. 2012;105(11):1083–8. doi: 10.1093/qjmed/hcs138 22855285

[18] Van Ditshuizen JC, Sewalt CA, Palmer CS, Van Lieshout EMM, Verhofstad MHJ, Den Hartog D, et al. The definition of major trauma using different revisions of the abbreviated injury scale. Scand J Trauma Resusc Emerg Med. 2021;29(1):71. doi: 10.1186/s13049-021-00873-7 34044857 PMC8162011

[19] Schluter PJ. The Trauma and Injury Severity Score (TRISS) revised. Injury. 2011;42(1):90–6. doi: 10.1016/j.injury.2010.08.040 20851394

[20] Moini M, Rezaishiraz H, Zarineh A, Rasouli M. Evaluation of quality of trauma care in a local hospital using a customization of ASCOT. Eur J Trauma Emerg Surg. 2009;35(1):56–60. 26814533 10.1007/s00068-008-7044-x

[21] Iqbal S, Mahgoub I, Du E, Leavitt M, Asghar W. Advances in healthcare wearable devices. NPJ Flex Electron. 2021;5(1):1–14.

[22] Heikenfeld J, Jajack A, Rogers J, Gutruf P, Tian L, Pan T, et al. Wearable sensors: modalities, challenges, and prospects. Lab Chip. 2018;18(2):217–48. doi: 10.1039/c7lc00914c 29182185 PMC5771841

[23] Seneviratne S, Hu Y, Nguyen T, Lan G, Khalifa S, Thilakarathna K, et al. A survey of wearable devices and challenges. IEEE Commun Surv Tutorials. 2017;19(4):2573–620. doi: 10.1109/comst.2017.2731979

[24] Majumder S, Mondal T, Deen MJ. Wearable sensors for remote health monitoring. Sensors (Basel). 2017;17(1):130. doi: 10.3390/s17010130 28085085 PMC5298703

[25] Kamišalić A, Fister I, Turkanović M, Karakatič S. Sensors and functionalities of non-invasive wrist-wearable devices: A review. Sensors. 2018;18(6):1714. 29799504 10.3390/s18061714 PMC6021794

[26] Kaewkannate K, Kim S. A comparison of wearable fitness devices. BMC Public Health. 2016;16:433. doi: 10.1186/s12889-016-3059-0 27220855 PMC4877805

[27] Hasan K, Biswas K, Ahmed K, Nafi N, Islam M. A comprehensive review of wireless body area network. J Network Comput Appl. 2019;143:178–98.

[28] Negra R, Jemili I, Belghith A. Wireless body area networks: applications and technologies. Proce Comput Sci. 2016;83:1274–81.

[29] Mosenia A, Sur-Kolay S, Raghunathan A, Jha NK. Wearable medical sensor-based system design: a survey. IEEE Trans Multi-Scale Comp Syst. 2017;3(2):124–38. doi: 10.1109/tmscs.2017.2675888

[30] Smith GB, Prytherch DR, Schmidt PE, Featherstone PI. Review and performance evaluation of aggregate weighted “track and trigger” systems. Resuscitation. 2008;77(2):170–9. doi: 10.1016/j.resuscitation.2007.12.004 18249483

[31] Breteler MJM, KleinJan EJ, Dohmen DAJ, Leenen LPH, van Hillegersberg R, Ruurda JP, et al. Vital signs monitoring with wearable sensors in high-risk surgical patients: a clinical validation study. Anesthesiology. 2020;132(3):424–39. doi: 10.1097/ALN.0000000000003029 31743149

[32] Xu H, Li P, Yang Z, Liu X, Wang Z, Yan W, et al. Construction and application of a medical-grade wireless monitoring system for physiological signals at general wards. J Med Syst. 2020;44(10):182. doi: 10.1007/s10916-020-01653-z 32885290 PMC7471584

[33] Bansal A, Joshi R. Portable out-of-hospital electrocardiography: a review of current technologies. J Arrhythm. 2018;34(2):129–38. doi: 10.1002/joa3.12035 29657588 PMC5891427

[34] Ferri J, Llinares R, Segarra I, Cebrián A, Garcia-Breijo E, Millet J. A new method for manufacturing dry electrodes on textiles. validation for wearable ECG monitoring. Electrochem Commun. 2022;136:107244.

[35] Vandenberk T, Stans J, Mortelmans C, Van Haelst R, Van Schelvergem G, Pelckmans C, et al. Clinical validation of heart rate apps: mixed-methods evaluation study. JMIR Mhealth Uhealth. 2017;5(8):e129. doi: 10.2196/mhealth.7254 28842392 PMC5591405

[36] Ghamari M. A review on wearable photoplethysmography sensors and their potential future applications in health care. Int J Biosens Bioelectron [Internet]. 2018 [cited 2023 Mar 10];4(4). Available from: https://medcraveonline.com/IJBSBE/a-review-on-wearable-photoplethysmography-sensors-and-their-potential-future-applications-in-health-care.html 10.15406/ijbsbe.2018.04.00125 PMC6426305 30906922

[37] Chan ED, Chan MM, Chan MM. Pulse oximetry: understanding its basic principles facilitates appreciation of its limitations. Respir Med. 2013;107(6):789–99. doi: 10.1016/j.rmed.2013.02.004 23490227

[38] Charlton P, Bonnici T, Tarassenko L, Alastruey J, Clifton D, Beale R. Extraction of respiratory signals from the electrocardiogram and photoplethysmogram: technical and physiological determinants. Physiol Measure. 2017;38(5):669–90. 10.1088/1361-6579/aa670e 28296645

[39] Rahman M, Morshed BI. Extraction of respiration rate from wrist ECG signals. In: 2021 IEEE 12th Annual Ubiquitous Computing, Electronics & Mobile Communication Conference (UEMCON). 2021. p. 0565–70.

[40] Hernandez J, McDuff D, Picard R. BioWatch: estimation of heart and breathing rates from wrist motions. Vol. 1. EAI Endorsed Transactions on Pervasive Health and Technology; 2015.

[41] Vanegas E, Igual R, Plaza I. Sensing systems for respiration monitoring: a technical systematic review. Sensors (Basel). 2020;20(18):5446. doi: 10.3390/s20185446 32972028 PMC7570710

[42] Niswar M, Wijaya AS, Ridwan M, Adnan, Ilham AA, Sadjad RS, et al. The design of wearable medical device for triaging disaster casualties in developing countries. In: 2015 Fifth International Conference on Digital Information Processing and Communications (ICDIPC). 2015. p. 207–12.

[43] Kahn C, Schultz C, Miller K, Anderson C. Does START triage work? An outcomes assessment after a disaster. Ann Emerg Med. 2009;54(3):424–30.e1. 19195739 10.1016/j.annemergmed.2008.12.035

[44] Chang C, Murphy R. Towards robot-assisted mass-casualty triage. 2007. p. 267.

[45] Çalhan A, Cicioğlu M, Ceylan A. EHealth monitoring testbed with fuzzy based early warning score system. Comput Methods Programs Biomed. 2021;202:106008. doi: 10.1016/j.cmpb.2021.106008 33640651

[46] Posada-Quintero HF, Chon KH. Innovations in electrodermal activity data collection and signal processing: a systematic review. Sensors (Basel). 2020;20(2):479. doi: 10.3390/s20020479 31952141 PMC7014446

[47] Desai U, Shetty AD. Electrodermal activity (EDA) for treatment of neurological and psychiatric disorder patients: a review. In: 2021 7th International Conference on Advanced Computing and Communication Systems (ICACCS). 2021. p. 1424–30.

[48] Lima R, Osório D, Gamboa H. Heart rate variability and electrodermal activity biosignal processing: predicting the autonomous nervous system response in mental stress. In: Roque A, Tomczyk A, De Maria E, Putze F, Moucek R, Fred A, et al., editors. Biomedical engineering systems and technologies. Cham: Springer International Publishing; 2020. p. 328–51.

[49] Shaffer F, Ginsberg J. An overview of heart rate variability metrics and norms. Front Public Health. 2017;5. 10.3389/fpubh.2017.00258 PMC5624990 29034226

[50] Zia J, Kimball J, Rolfes C, Hahn J-O, Inan OT. Enabling the assessment of trauma-induced hemorrhage via smart wearable systems. Sci Adv. 2020;6(30):eabb1708. doi: 10.1126/sciadv.abb1708 32766449 PMC7375804

[51] Convertino V, Schauer S, Weitzel E, Cardin S, Stackle M, Talley M, et al. Wearable sensors incorporating compensatory reserve measurement for advancing physiological monitoring in critically injured trauma patients. Sensors. 2020;20(22):1–12. doi: 10.3390/s202205678 PMC7697670 33182638

