# Supplementary figures and images for: A new trauma severity scoring system adapted to wearable monitoring: A pilot study
Source: PLoS One. 2025 Mar 4;20(3):e0318290. doi: 10.1371/journal.pone.0318290 (PMC11878944; doi:10.1371/journal.pone.0318290)

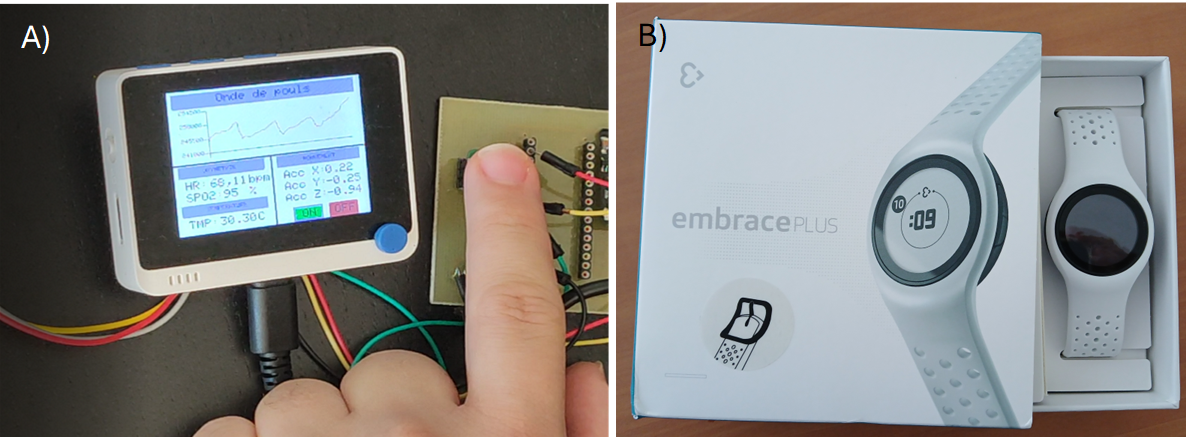

Supplement: S1 Fig — (TIF) [file pone.0318290.s001.tif]
