# Supplementary material for: A new trauma severity scoring system adapted to wearable monitoring: A pilot study
Source: PLoS One. 2025 Mar 4;20(3):e0318290. doi: 10.1371/journal.pone.0318290 (PMC11878944; doi:10.1371/journal.pone.0318290)
Supplement: S1 File — (DOCX) [file pone.0318290.s002.docx]

S2 Appendix

# Survey conducted to determine intermediate scores’ boundary values

The survey to determine intermediate scores’ boundary values has been disseminated through the French Society of Anaesthesia and Reanimation (Société Française d’Anesthésie et de Réanimation). For each of the three physiological parameters (heart rate, respiratory rate, SpO_2_), expert health professionals have been asked to indicate the boundary values that they would use to separate normal, abnormal and critical ranges of values for an adult subject, as illustrated on Fig S2A.


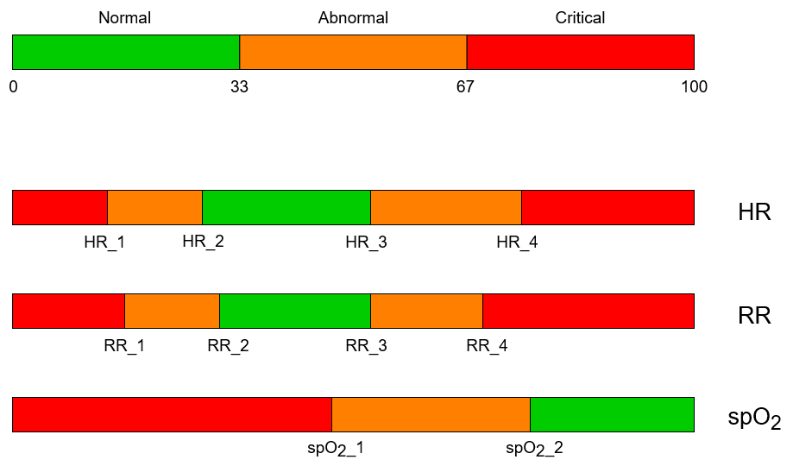


**Fig S2A. Boundaries of normal, abnormal, and critical value ranges for heart rate (HR), respiratory rate (RR), and blood oxygenation (SpO_2_).** This figure has been used in the survey conducted among expert health professionals. S_H_(HR_1)=67; S_H_(HR_2)=33; S_H_(HR_3)=33; S_H_(HR_4)=67; S_R_(RR_1)=67; S_R_(RR_2)=33; S_R_(RR_3)=33; S_R_(RR_4)=67; S_O_(SpO_2__1)=67; S_O_(SpO_2__4)=33.

Answers from 54 health professionals have been received in total (29 emergency doctors, 24 anaesthetist-intensive care doctors, and 1 paediatric nurse). They were junior (0-3 years) for 18.5 %, intermediate (3-10 years) for 40.7 % and senior (more than 10 years) for 40.7 %. No answer variation related to specialty or experience level has been noticed. Thus, all the answers were considered together. The results are presented in Table S2A and Fig S2B.

Table S2A and Fig S2B show that the answers to the survey are consistent with each other. The standard deviation of the answers for heart rate boundary values varies from below 10 bpm for the lowest value HR_1 to 16 bpm for HR_4. For respiratory rate, it ranges from 2 rpm for the lowest RR_1 to 5 rpm for RR_4. Regarding SpO_2_, the standard deviation is below 4 %. For simplicity, the median values are used as boundaries between normal, abnormal, and critical value ranges of HR, RR, and SpO_2_.

**Table S2A. Average, median, and standard deviation of boundaries between normal, abnormal, and critical value ranges for heart rate (HR), respiratory rate (RR), and peripherical oxygen saturation (SpO_2_) according to the survey results.** The boundaries notations are related to Fig. S2A.

|  | Mean | Median | Standard deviation |
| --- | --- | --- | --- |
| HR_1 (bpm) | 39,9 | 40 | 9,1 |
| HR_2 (bpm) | 57,8 | 60 | 9,3 |
| HR_3 (bpm) | 98,9 | 100 | 11,2 |
| HR_4 (bpm) | 124,8 | 122,5 | 16,2 |
| RR_1 (rpm) | 8,4 | 8 | 1,9 |
| RR_2 (rpm) | 12,6 | 12 | 2,3 |
| RR_3 (rpm) | 21,4 | 20 | 3,7 |
| RR_4 (rpm) | 29,3 | 30 | 5,2 |
| spO2_1 (%) | 86,4 | 85 | 3,4 |
| spO2_2 (%) | 92,8 | 93,5 | 2,4 |


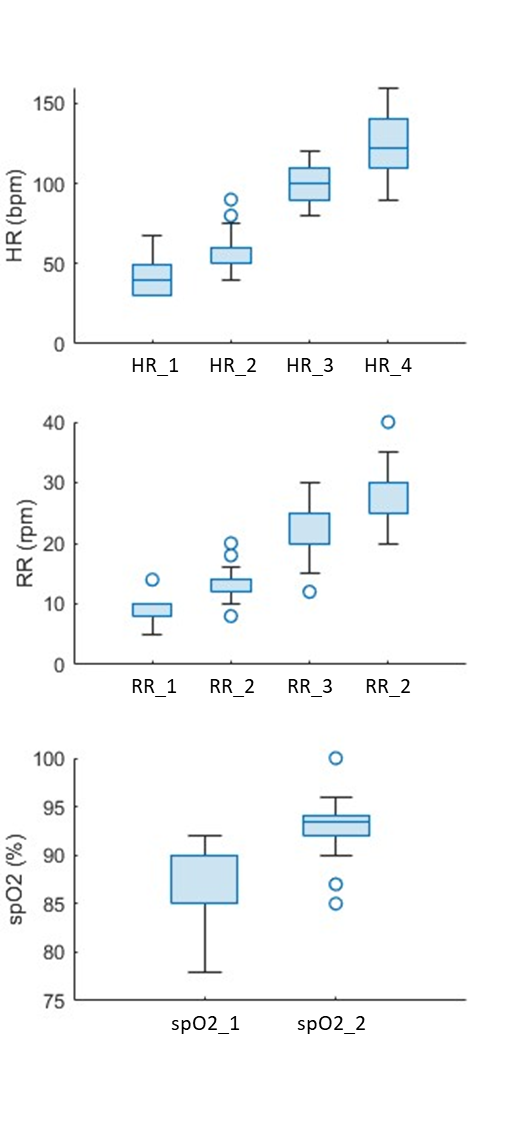


**Fig S2B. Boxplots of boundaries between normal, abnormal, and critical value ranges for heart rate (HR), respiratory rate (RR), and peripherical oxygen saturation (SpO_2_) according to the survey results.** The notation of the boundary values is in accordance with Fig S2A.
