# Supplementary material for: A new trauma severity scoring system adapted to wearable monitoring: A pilot study
Source: PLoS One. 2025 Mar 4;20(3):e0318290. doi: 10.1371/journal.pone.0318290 (PMC11878944; doi:10.1371/journal.pone.0318290)
Supplement: S2 File — (DOCX) [file pone.0318290.s003.docx]

S3 Appendix

# Merging function: class’ number of combinations

The number of possible combinations leading to green, orange, or red output in relation with Figure 1 are here calculated. In the case of a n-$n$combination with repetition {C_1_,C_2_,…C_n_ } of the set {0,1,2}, the number N_tot_ of possible combinations is the following (Equation S1):

|  | $N_{tot}=N_{G}+N_{O}+N_{R}=3^{n}$ | ( S1 ) |
| --- | --- | --- |

Where N_G_, N_O_, N_R_ are respectively the number of possible combinations leading to a green, orange, or red output according to condition (i) of 2.1.3.

Equiprobability is assumed between the three set elements (Equation S2).

|  | $p\left( 0 \right)=p\left( 1 \right)=p\left( 2 \right)=\frac{1}{3}$ | ( S2 ) |
| --- | --- | --- |

In the case n=3 and according to Figure 1, S_HRO_≤33 if and only if [C_H_,C_R_,C_O_]=[0,0,0]. The only combination leading to a green output, is the one in which all elements of the code array equal 0 (Equation S3).

|  | $N_{G}=1$ | ( S3 ) |
| --- | --- | --- |

Then, 33<S_HRO_<67 is equivalent to “∀i∈{H,R,O},C_i_≠2 and {C_H_,C_R_,C_O_}≠ {0,0,0} “. The output is orange if no element of the code array equal 2 and at least one of the elements is different from 0. If the probability to get a red is considered as a success, the probability to have 0 success is expressed as in Equation S4.

|  | $P\left( X=k \right)=\binom{n}{k}{p\left( 2 \right)}^{k}\left( 1-p\left( 2 \right) \right)^{n-k}$  $P\left( X=0 \right)=\frac{n!}{0!\left( n-0 \right)!}\left( \frac{1}{3} \right)^{0}\left( 1-\frac{1}{3} \right)^{n-0}=\left( \frac{2}{3} \right)^{n}$ | ( S4 ) |
| --- | --- | --- |

Therefore, the number of possible combinations leading to an orange output is calculated in Equation S5.

|  | $N_{O}=P\left( X=0 \right)*N_{tot}-N_{G}=\left( \frac{2}{3} \right)^{n}*3^{n}-1=2^{n}-1$ | ( S5 ) |
| --- | --- | --- |

The number of red combinations can finally be deducted (Equation S6).

|  | $N_{R}=N_{tot}-N_{G}+N_{O}=3^{n}-2^{n}$ | ( S6 ) |
| --- | --- | --- |
