# Supplementary material for: A new trauma severity scoring system adapted to wearable monitoring: A pilot study
Source: PLoS One. 2025 Mar 4;20(3):e0318290. doi: 10.1371/journal.pone.0318290 (PMC11878944; doi:10.1371/journal.pone.0318290)
Supplement: S3 File — (DOCX) [file pone.0318290.s004.docx]

S4 Appendix

# Colour limit thresholds’ optimisation

To determine a subject’s score colour, the thresholds T_GO_=33 and T_OR_=67 have been used by default as green/orange and orange/red limits. As an attempt to improve the prediction success rate, optimised thresholds have been determined graphically by plotting the Receiver Operating Characteristic (ROC) curves (Fig S4A). This has been performed thanks to Matlab’s *perfcurve* function. The optimised thresholds, as well as the prediction success rates when using these optimised thresholds, are given in Table S4A.


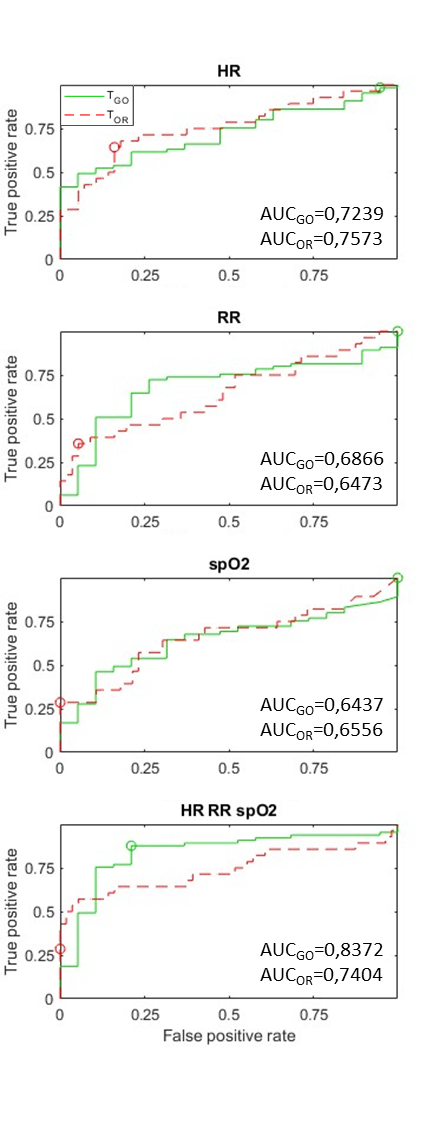


**Fig S4A. Receiver Operating Characteristic curve of subject’s colour code prediction with the score S_H_, S_R_, S_O_ and S_HRO_.** Green continuous line and red dashed line correspond respectively to the green/orange (GO) and to the orange/red (OR) separations. The points corresponding to the optimal threshold are indicated; and the areas under curves AUC_GO_ and AUC_OR_ are given.

**Table S4A. Optimised colour limit thresholds T_GO_ and T_OR_ determined graphically by plotting the Receiver Operating Characteristic and corresponding prediction success rate PRS.**

|  | T_GO_ | T_OR_ | PRS (%) |
| --- | --- | --- | --- |
| S_H_ | 20 | 34 | 54 |
| S_R_ | 26 | 56 | 52 |
| S_O_ | 13 | 53 | 49 |
| S_HRO_ | 34 | 71 | 73 |

The threshold values are especially different from 33 and 67 for heart rate and SpO_2_. Compared to the prediction success rates in Table 4, the prediction success rates of the intermediate score S_H_ and S_O_ value are 54 % and 49 % respectively, compared to 38 % and 36 % when using 33 and 67 as thresholds. The prediction success rates without or with the threshold optimisation are the same for the respiratory rate. Regarding the global score, the prediction success rates are more or less the same without or with the optimisation. Therefore, the threshold optimisation has been effective for the intermediate scores (except S_R_), but there is no significant improvement for the global score.

In order to optimise the global score, each intermediate score has been normalised with a linear function n_P_(S_P_)=a_P_*S_P_+b_P_ with P∈{H,R,O} so that n(T_GO_)=33 and n(T_OR_)=67. However, the resulting prediction score rate of the global score function is only 56 %. The corresponding confusion matrix is given in Fig S4B. It is noted that no subject is predicted anymore as green. The normalisation results in raising the intermediate scores (in relation with the fact that the minimum value for the intermediate functions is not zero, as illustrated Fig 3), which is not adapted when considering the three physiological parameters together.


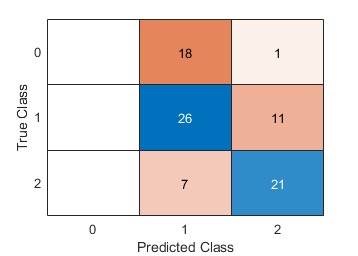


**Fig S4B. Confusion matrix of colour code prediction using the optimised scoring system (e.g. calculated with the normalised intermediate scores**). 0 : green, 1: orange, 2: red
